# Cerebral Hæmorrhage.—II
*A Lecture delivered at the London School of Clinical Medicine.


**Published:** 1912-04-20

**Authors:** Guthrie Rankin

**Affiliations:** Physician to the "Dreadnought" and Royal Waterloo Hospitals.


### CEREBRAL HÆMORRHAGE.—II.*

By GUTHRIE RANKIN, M.D. Glas., F.R.C.P. Lond. and Edin., Physician to the "Dreadnought" and Royal Waterloo Hospitals.

The consequent paralysis varies according to the extent and situation of the cerebral damage. The favourite seat of vascular disturbance is, as has been already pointed out, the lenticulostriate branch of the middle cerebral artery; the most common variety of paralysis therefore is a hemiplegia, which affects the side of the body opposite to the site of the lesion in the brain. The left hemisphere of the brain is more frequently involved than the right, so that, in the majority of cases, the hemiplegia is right-sided and accompanied by more or less aphasia. Hemiplegia may result from a lesion situated anywhere between the cortex and the decussation of the pyramids in the medulla. If the motor tract is disturbed only, as when it is compressed from without, the paralysis will probably be temporary, but, if it is structurally damaged, the paralysis must be, to a greater or less extent, permanent. Sometimes hemi-anæsthesia is associated with hemiplegia and, when at all well marked or lasting, indicates such a situation of the hæmorrhage as to implicate the hinder part of the posterior segment of the capsule. It is more frequently met with in cases of softening than in hæmorrhage. If the hæmorrhage has occurred at a situation above the crus cerebri, the consequent paralysis affects the tongue, the lower facial muscles, and those of the arm and leg on the opposite side of the body, because all the fibres decussate below the seat of lesion. The upper facial muscles are not affected, and Broadbent's explanation, which is generally accepted, is that muscles, like the occipito-frontalis, though morphologically bilateral, have through long association become equally well innervated from either hemisphere. There is, in most cases, evidence of the bilateral representation of their movement in the want of power of independent closure of the eye on the paralysed side. A person who, before the attack, could shut either eye independently of the other, is found, after it, unable to close that on the paralysed side alone.

### Crossed Hemiplegia.

If the morbid change is situated either in the crus or in the pons, or in the medulla, a condition of crossed hemiplegia ensues in which there is paralysis of one or more cranial nerves on one side of the body accompanying the ordinary form of hemiplegia on the other. Under such circumstances, the implicated cranial nerves are situated on the same side as the lesion in which they are involved at a level below that of their decussation.

In pontine hæmorrhage the initial loss of consciousness is rapid and profound, should the effusion of blood be large. There is complete flaccid paralysis with abolition of the reflexes, the pupils are contracted to the smallest size, respiration is often markedly irregular. Paralysis or paresis of the facial or of the fifth cranial nerves will be noted on the side corresponding to the lesion in cases where the effusion is more moderate.

If the hæmorrhage has occurred in the crus the hemiplegia is accompanied by occulo-motor paralysis on the side of the lesion.

### Evidences of Local Irritation.

Within about forty-eight hours of the seizure, the irritation caused by the hæmorrhage gives rise to headache, sometimes to slight delirium, and to twitchings and rigidity in the paralysed limbs. In favourable cases, these evidences of local irritation subside rapidly, but if the hæmorrhage is more serious, trophic changes—especially bedsores—may ensue, and other evidences may be forthcoming to arouse suspicions of the onset of cerebral softening at the seat of lesion. There is always some—often very slight—change left in the patient's mental condition; it may be so unimportant as to escape passing notice, but the patient is himself aware of intellectual impairment and frequently experiences an emotional instability which is an entirely new feature of his temperament and one which, on account of its uncontrollability, greatly distresses him. Unless the area of damage has been very small, recovery of muscular power is seldom quite complete. The leg recovers more speedily and perfectly than the arm in most cases. Ultimate rigidity of the paralysed limbs from secondary sclerosis of the lateral columns is to be looked for, and every care must be taken to minimise the risk of permanent contractures. In addition to the ordinary apoplexy which has just been described, there are four other varieties of the condition to which passing reference must be made:—

(*a*) Simple cases in which no lesion is found post-mortem except a general œdema of the brain, giving rise presumably to a serous apoplexy.

(*b*) Ingravescent cases in which there is a very gradual onset, followed, after some hours, by loss of power and drowsiness which deepens into coma and often ends in death.

The leakage in such cases from the ruptured vessel is gradual but progressive:—

(*c*) Fulminant cases in which there is an extensive extravasation of blood, and death ensues within a few minutes. The coma is complete and sudden and is accompanied by general paralysis or by convulsions, by irregular breathing, slow and intermittent pulse, cyanosis, and clammy, cold skin. The movements of respiration and the cardiac pulsations are the only outward evidences of life.

(*d*) Meningeal cases in which the onset is sudden and severe and is often preceded by headache, vomiting, muscular twitchings, or even convulsions. There is an absence of hemiplegia or regional distribution of paralysis. If the hæmorrhage is at all

* A Lecture delivered at the London School of Clinical Medicine.

severe, the attack is usually fatal within forty-eight hours. The most frequent cause is injury, but it may ensue upon rupture of an aneurysm situated on one of the larger arteries at the base of the brain, and it may be also a sequel to acute rheumatism or occur during the puerperium. It is met with in newly-born children, especially after a difficult or instrumental delivery, and is the forerunner of subsequent infantile spastic paraplegia. Vascular degeneration is not a frequent cause of hæmorrhage in this situation.

In cerebral hæmorrhage the immediate prognosis is always doubtful; it depends upon the situation of the lesion, and the intensity of the symptoms. If the localising symptoms suggest the pons or medulla as the site of hæmorrhage, the outlook is extremely grave. If there is complete coma lasting over twenty-four hours, recovery is always doubtful. Cheyne-Stokes respiration persisting for more than six hours is ominous. A comparatively early return to consciousness followed by a sudden relapse into a comatose state suggests secondary ventricular hæmorrhage and is of dangerous import. If the subsequent rise of temperature, after the initial fall, does not exceed 102°, recovery will probably ensue even if the temperature persists about this level for twenty-four hours, but if, within a few hours of the initial fall, the temperature rises to 104° or more, an early fatal issue is almost certain.

Until the expiry of some weeks it is difficult to be at all certain as to the permanent amount of physical or mental defect that may remain, but, speaking generally, at the end of a month the maximum of recovery is attained, though it is, even then, too soon to know what secondary degenerative changes may ensue or how far they may go.

When a patient is first seen in an unconscious condition it may be possible to arrive at a positive opinion as to causation. Prima facie a history of a blow or fall on the head, a record of specific disease, or the existence of pronounced atheroma in a person of advanced years, would be strong evidence in favour of an intracranial lesion. A history of the unconsciousness having come on suddenly, or of having developed gradually after previous complaint of headache, vertigo, or sickness would afford further confirmation of a cerebral hypothesis. The existence of greater muscular flaccidity on one side of the body compared with the other, of a Babinsky response in the foot of the flaccid side, of a higher temperature on that than on the other side, would practically determine the lesion as being intracranial, while the state of the pupils, pulse, respiration and urine would add minor links to the chain of diagnostic evidence. But assuming it to be clear that the condition is one of apoplexy, a further point of difficulty arises in coming to a conclusion as to whether the lesion is a hæmorrhage, or whether it arises from either of the two remaining vascular disturbances from which hæmorrhage may, in many instances, be so easily mistaken, viz. thrombosis or embolism.

The following differential points are useful aids to a correct solution of the problem.

(*a*) Hæmorrhage generally comes on abruptly in a person over fifty years of age, whose vessels present evidence of atheromatous change, and whose pulse is of high tension. Albuminuria, low specific gravity of the urine, and hypertrophy of the left ventricle are contributory signs of confirmatory value. The coma is profound in proportion to the extent of hæmorrhage. The temperature is at first lowered, but rises within forty-eight hours in all cases except such as prove fatal within that time.

(*b*) Thrombosis is usually of gradual onset and is preceded by premonitory symptoms, such as vertigo, localised formication or numbness, mental dulness, and headache. It is unaccompanied, at its onset, by loss of consciousness and it may occur at any age. The sequential paralysis is of gradual development and is liable to remissions and exacerbations. There is frequently a history of syphilis.

(*c*) Embolism causes loss of consciousness proportionate to the size of vessel blocked, and is of sudden onset. It mostly occurs in persons under forty years of age and is especially frequent in women. It is always associated with aneurysm or valvular disease of the heart, and is particularly frequent in cases where the mitral valve is the seat of old-standing sclerotic change. There is no initial depression of temperature as in hæmorrhagic cases, but within forty-eight hours there is a temperature rise consequent upon reactionary changes at the seat of lesion.

The conditions with which cerebral hæmorrhage is most apt to be mistaken are:—

Uræmia: In this condition the coma is not, as a rule, so profound as in apoplexy. The patient may be roused sufficiently to answer questions, but immediately relapses into his previous drowsy state. There is no paralysis, but convulsions are frequent and of general distribution. The temperature is persistently depressed and does not become febrile; the pulse is small, hard, and of plus tension; and the urine is deficient in urea and contains albumen. The breathing is "hissing" rather than stertorous in character; and the odour of the breath is ammoniacal. There is often a history of previous œdema, and the retinæ may present the usual degenerative changes found in association with Bright's disease.

Alcoholism: The coma is less profound in alcoholism than in apoplexy, and, when a history can be obtained, there is a record of excessive indulgence, particularly in ardent spirits, with previous boisterous excitement. The breath smells of alcohol, but too much stress must not be laid upon this one point. The temperature is subnormal; the pulse soft and often irregular; the features bloated and swollen but symmetrical; the skin warm and moist; and, as a rule, there is an absence of localised paralysis or convulsion. The pupils are firmly contracted when the patient is at rest, but dilate temporarily when he is roused. Examination of the contents of the stomach often throws light upon a doubtful case, and confirmation is sometimes forthcoming in a subsequent attack of unmistakable delirium tremens.

(*To be continued.*)

